# Role of Body Composition in the Prediction of Skeletal Fragility Induced by Hormone Deprivation Therapies in Cancer Patients

**DOI:** 10.1007/s11912-023-01447-9

**Published:** 2023-08-25

**Authors:** Alberto Dalla Volta, Irene Caramella, Pierluigi Di Mauro, Marco Bergamini, Deborah Cosentini, Francesca Valcamonico, Carlo Cappelli, Marta Laganà, Nunzia Di Meo, Davide Farina, Rebecca Pedersini, Gherardo Mazziotti, Alfredo Berruti

**Affiliations:** 1 Department of Medical and Surgical Specialties, Radiological Sciences, and Public Health, Medical Oncology Unit, University of Brescia, Azienda Socio Sanitaria Territoriale (ASST) Spedali Civili, 25123 Brescia, Italy; 2 Department of Experimental Sciences, Unit of Endocrinology and Metabolism, University of Brescia, Azienda Socio Sanitaria Territoriale (ASST) Spedali Civili, Brescia, Italy; 3 Department of Medical and Surgical Specialties, Radiological Sciences, and Public Health, Radiology Unit, University of Brescia, Azienda Socio Sanitaria Territoriale (ASST) Spedali Civili, Brescia, Italy; 4 Breast Unit, Azienda Socio Sanitaria Territoriale (ASST) Spedali Civili, Brescia, Italy; 5 Department of Biomedical Sciences, Humanitas University, Pieve Emanuele, Milan, Italy; 6 Endocrinology, Diabetology and Andrology Unit, IRCCS Humanitas Research Hospital, Rozzano, Milan, Italy

**Keywords:** Sarcopenic obesity, Hormonal deprivation, Body composition, Bone health, Prostate cancer, Breast cancer

## Abstract

**Purpose of Review:**

This review paper is intended to show that changes in body composition are key in the pathogenesis of bone fragility amongst patients with breast and prostate cancer receiving hormone deprivation therapies (HDTs) and that the mechanism is based on the development of alterations in bone quality rather than in bone quantity.

**Recent Findings:**

Preclinical and clinical data suggest a tight connection amongst bone, adipose and muscular tissues by means of several soluble mediators, potentially leading to (1) bone resorption and bone quality deterioration in sarcopenic obese subjects, (2) bone mineral deposition in healthy trained subjects. Cancer patients treated with HDTs frequently fall into the first condition, named osteosarcopenic obesity.

**Summary:**

Current clinical guidelines for the prevention of treatment-induced osteoporosis focus on bone mineral density (BMD) as a main predictive factor for fracture risk; however, the pathophysiology underlying HDT-induced bone fragility differs from that of primary and postmenopausal osteoporosis, suggesting a prevalent role for bone quality alterations. Focusing on available data from clinical trials, in our review we suggest osteosarcopenic obesity as a common target for the prevention and treatment of HDTs-related metabolic and skeletal complications, beyond a BMD-centred approach.

## Introduction

Androgen deprivation therapy (ADT) by luteinizing hormone releasing hormone agonists or antagonists is the mainstay of treatment of men with progressive prostate cancer, in addition to next generation hormonal agents (NGHAs), chemotherapy or targeted agents [1]. Oestrogen deprivation with aromatase inhibitors (AIs) is a reference therapeutic approach for post-menopausal women with breast cancer either in adjuvant or metastatic setting [2,3].

Both hormone deprivation therapies (HDTs) lead to profound alterations in the hormonal milieu. In men with prostate cancer, ADT is associated with a deep fall of testosterone serum levels and a reduction of oestrogen levels by lacking the substrate for aromatase [4]. In postmenopausal women, aromatase inhibition causes depletion of oestrogen levels, which may be associated to increased androgens. Hormonal derangements induced by HDTs cause bone metabolism alterations, both in men with prostate cancer and in women with breast cancer, resulting in bone fragility and increased risk of fractures [5,6]. The pathophysiology underlying HDT-induced bone fragility has not been fully elucidated [7••]. Both ADT and AIs are known to significantly lower bone mineral density (BMD), a measure of bone quantity, which is recognized as an independent predictor of fracture risk in postmenopausal osteoporosis [8]. BMD is also recommended as a clinical variable inside FRAX algorithm for the decision making regarding the use of bone protecting agents (e.g. bisphosphonates or denosumab) in non-metastatic cancer patients receiving HDTs [9]. However, there is consistent evidence that BMD may not be a reliable predictive tool for fracture risk assessment in this clinical setting [7••].

ABCSG-18 study, a large randomized clinical trial which enrolled patients with early breast cancer treated with adjuvant AIs, showed that denosumab, a RANK-L inhibitor, reduces the incidence of new clinical and vertebral fractures (VFs) [10]. Noteworthy, the cumulative proportion of fractures in the placebo arm of this study was not different when comparing patients with low BMD to those with normal BMD [10].

In a single-centre cross-sectional study that recruited postmenopausal women with early breast cancer, whether or not treated with AIs, BMD was significantly associated with the prevalence of VFs in untreated but not in treated patients [11].

As to prostate cancer patients, another cross-sectional study, recruiting men with non-metastatic disease, receiving ADT for at least 6 months, showed that the use of BMD (spine and hip) alone resulted in the misdiagnosis of approximately 75% of patients with clinically defined osteoporosis (e.g. the presence of VFs at morphometric analysis) [12].

Finally, in a real-life clinical practice study, which investigated the accuracy of FRAX algorithm for the prediction of VFs in breast and prostate cancer patients receiving HDTs, BMD was shown to be associated with morphometric vertebral fractures at a more restrictive cutoff (i.e. −1.0 SD) compared to the recommended reference value (i.e. −2.5 SD) [13•].

Taken together, these data qualify BMD as a weak predictor for fracture risk in the context of secondary osteoporosis, despite bone loss is a common side effect during HDTs. This apparent contradiction can be explained inferring a potential prevalent role for bone quality alterations rather than bone loss in the development of HDTs induced bone fragility. Bone quality is a complex issue and refers to the multifaceted properties of bone tissue (including bone microarchitecture, geometry, turnover and composition of bone matrix) contributing, together with BMD, to determine fracture risk [14,15].

Over the recent years, many dual-energy X-ray absorptiometry (DXA)-based tools have been developed to explore skeletal features of bone quality and to refine fracture risk prediction. As a matter of fact, trabecular bone score (TBS), a texture parameter coming from lumbar spine DXA images and reflecting bone microarchitecture (i.e. bone quality), was shown to decrease early during HDTs independently of BMD [16].

Even though both postmenopausal and HDTs related bone fragility are associated with a drop of sexual hormones, a differential pathophysiology is required to explain why BMD is no longer the most relevant predictor for fracture in the latter condition, where bone quality alterations seem to prevail.

Noteworthy, besides measurement of BMD and bone quality parameters, DXA is a non-invasive tool to measure reliably body composition alterations, which frequently result as an effect of HDTs. The relationship between body composition and bone metabolism is an emerging area of research and clinical interest [17]. As a matter of fact, recent studies provided consistent evidence that increase in adiposity and decrease in lean body mass might induce detrimental effects on bone in subjects exposed to HDTs [18•,^19^].

In this narrative review we dissected the available evidence supporting the role of body composition as a clinical variable implied in bone fragility and bone quality impairments amongst prostate and breast cancer patients on HDTs.

## Interactions Between Adipose Tissue and Bone

**Fig. 1:**
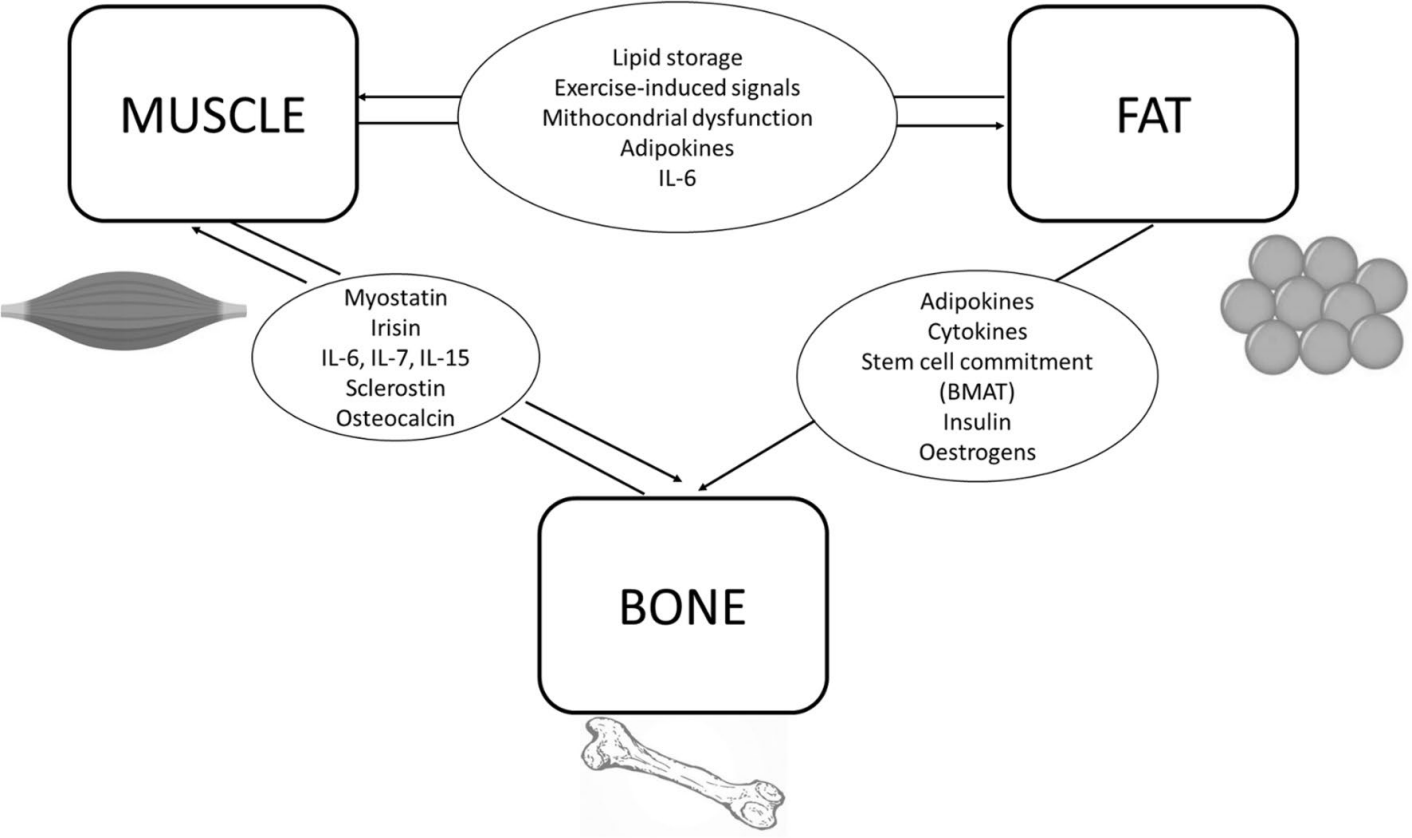
The complex interplay between muscle, fat and bone. BMAT bone marrow adipose tissue

Adipose tissue is progressively emerging as a modulator of whole-body homeostasis, including bone metabolism. Fat-bone interplay is mediated by several mechanisms: (1) secretion of peptides named adipokines with paracrine and endocrine activity, (2) proinflammatory cytokines, (3) differentiation of common cell precursors [20] (Fig. 1).

Amongst the adipokines, a key role is played by leptin and adiponectin. Leptin, mainly known as one of the principal endocrine regulators of appetite and energy expenditure, has also been shown to have effects on bone turnover by inhibiting the deposition of new mineralized mass. Counterwise, adiponectin supports bone mass formation by enhancing osteoblasts proliferation, migration and survival, whilst concomitantly limiting osteoclasts maturation. A clinical validation of the differential roles of these adipokines can be found amongst obese patients, where high levels of leptin and low levels of adiponectin result in a significant depletion of bone mass [21].

Another hormonal regulation of fat-bone metabolism is mediated by insulin. Insulin acts as a primary regulator of adipose tissue, both from a metabolic point of view (e.g. glucose cellular uptake) as well as altering fat-bone interplay. As a matter of fact, insulin resistance affects both adipose and bone metabolism: compensatory hyperinsulinemia acts on adipokine levels, induces an inflammatory milieu via the attraction of macrophages and stimulates bone resorption. In detail, it has been shown that insulin reduces osteoblast-mediated expression of osteoprotegerin, allowing receptor activation of nuclear factor-kB (NF-kB) ligand to enhance osteoclastic activity with consequent bone resorption [20].

Induction and maintenance of low-grade chronic inflammation by pro-inflammatory cytokines, locally produced in the adipose tissue, is another proposed pathway for bone metabolism derangement in obese subjects [22]. High levels of cytokines such as interleukin-6 (IL-6), interleukin-1 (IL-1) and tumour necrosis factor-alpha (TNF-α) are found amongst subjects with insulin resistance and obesity [23,24]. Long-term maintenance of this condition results in enhanced osteoclast differentiation (IL-6, IL-1, TNF- α) and suppression of osteoprotegerin production (TNF-α), eventually causing bone loss [25].

Finally, studies on the interplay between bone marrow adipose tissue (BMAT) and bone remodelling offer a unique opportunity to understand the interaction between adipocytes, osteoblasts and osteoclasts inside the same microenvironment. It is well recognized that adipogenesis is tightly linked to osteogenesis in the bone marrow milieu, since osteoblasts and adipocytes share a common precursor (i.e. the MSC) [26]. Determination of MSC commitment towards either cell lineage is a finely tuned process, and several specific transcription factors (such as Runx2 and osterix for osteoblasts, and PPARγ for adipocytes) are involved. It has been shown that enhanced PPARγ activity results in decreased osteoblastic commitment and enhanced osteoclastic recruitment, suggesting direct correlation between adipogenesis and bone resorption [27]. Indeed, in clinical studies, greater BMAT is associated with lower BMD and increased rate of VFs, further corroborating the role of fat tissue as a potential risk factor for bone fragility [28].

In conclusion, the complex interplay between adipose and bone tissues can be elucidated assuming that an increase in fat mass causes an uncoupling process between bone resorption and deposition, finally leading to bone quality alterations. However, obesity is traditionally viewed as a protective factor for bone health because of well-established positive effects of mechanical loading and increased oestrogen production by action of aromatase enzyme expressed in adipocytes. Along this line a low BMI is known to be a strong independent predictor of fracture risk in postmenopausal osteoporosis [29].

The coexistence of these two opposing mechanisms—detrimental through deterioration of bone quality, protective through increase in BMD—is known as “obesity paradox” [30]. A practical demonstration of this assumption was provided by a large Korean study, involving thousands of men and women, who underwent DXA scan [31]. This study showed that BMI and weight correlated positively with BMD, but negatively with TBS, validating the hypothesis of simultaneous detrimental effect exerted by adipose tissue on bone quality and beneficial effect on bone quantity. The net consequence of obesity on bone health thus depends on the predominance of either impairment of bone microarchitecture or improvement in bone mineral mass. Noteworthy, the protective effect of obesity on fracture risk generally prevails during adulthood, whilst the negative effect may be more pronounced in the elderly, in whom the skeletal impact of estrogens produced by adipose tissue might not be relevant [30].

### Predictive Effect of Fat Body Mass on Bone Fragility Induced by HDTs in Cancer Patients

Patients treated with HDTs for breast and prostate cancer frequently encounter alterations in body composition similarly to women during menopause period and men after development of endogenous hypogonadism [32,33,34].

**Fig. 2:**
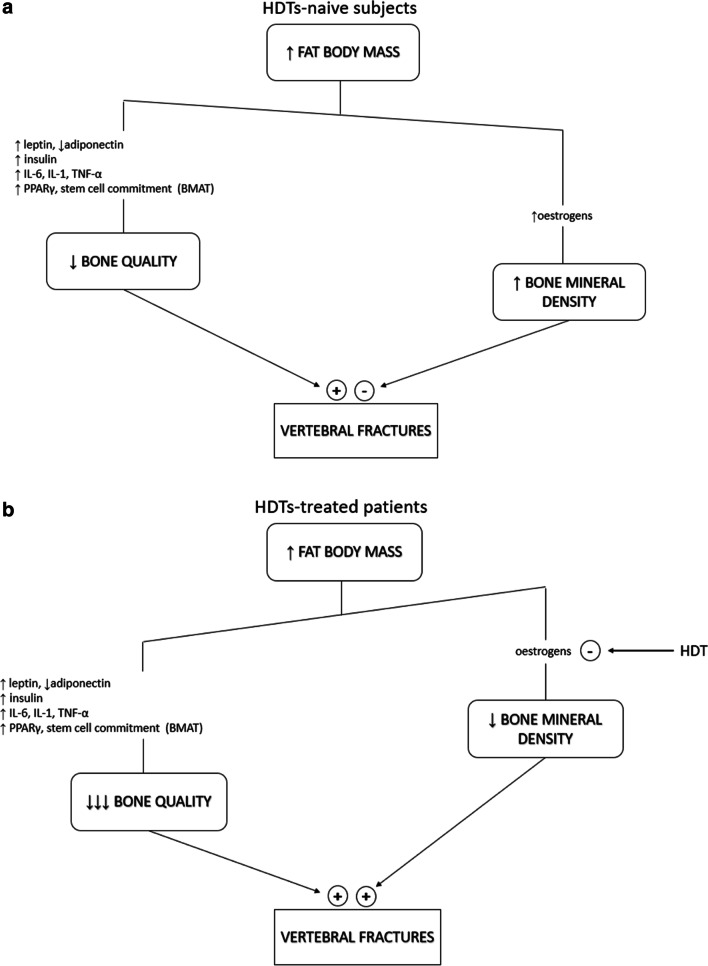
**A** In hormone deprivation therapies-naive subjects, fat mass counterbalances and usually overcomes the deleterious effect of adiposity on bone quality by means of preserved/increased bone mineral density through the increase in circulating levels of oestrogens. **B** As a result of hormonal deprivation therapies administration to obese patients, bone mineral density reduction adds to bone quality impairment causing a substantial increase in bone fragility. HDTs hormonal deprivation therapies, TNF-α tumour necrosis factor alpha, PPAR-γ peroxisome proliferator-activated receptor gamma, BMAT bone marrow adipose tissue

However, whilst in HDT-naive subjects fat mass counterbalances and usually overcomes the deleterious effect of adiposity by means of preserved/increased BMD (Fig. 2a), when HDTs are prescribed to obese patients or when obesity develops during HDTs, bone loss adds to bone quality impairment causing a substantial increase in bone fragility (Fig. 2b).

Therefore, obese patients could dramatically shift from a potentially protected condition to a high fracture risk when treated with HDTs (Fig. 2).

Some published evidence supports the aforementioned pathophysiological model.

A single-centre, cross-sectional study investigating the prevalence of VFs in a large series of postmenopausal women with early-stage breast cancer enrolled two different subgroups of patients which were either AI-naive or AI-treated for at least 2 years. The study results showed that amongst AI-naive subgroup VFs prevalence was higher in subjects presenting with fat body mass (FBM) below the median. Conversely, in AI-treated group the proportion of VFs showed an opposite pattern being higher in patients with FBM above the median. The apparently contradictory association of VFs prevalence with FBM values according to AI group was confirmed by multivariable analysis with a significant interaction test [19].

A retrospective longitudinal study enrolling non-metastatic prostate cancer patients showed that increased FBM upon ADT was associated with a higher risk of skeletal related events [35].

In another retrospective cross-sectional study, high BMI (>25 kg/m^2^) was a strong predictor of VFs in prostate cancer patients who were under ADT [13•]. However, in a small prospective study designed to evaluate changes in body composition, BMD and bone turnover markers before and after degarelix administration in non-metastatic prostate cancer, FBM did not correlate with either the markers of turnover at baseline conditions or with their changes after therapy [36•].

In summary, currently available evidence supports adiposity as a determinant factor of skeletal fragility in subjects under HDTs, suggesting that the use of BMI for the stratification of fracture risk in this clinical setting could be unreliable. This assumption is in marked contrast with international guidelines, which define higher BMI as a protective factor for fractures [9,37].

Due to the paucity and heterogeneity of the published series, the different parameters used to define adiposity and the different endpoints applied to assess bone fragility, the correlation between FBM and bone health deserves to be further studied in patients with breast and prostate cancer undergoing HDTs.

### Interactions Between Muscle and Bone

During the last decade, bone and muscle were increasingly recognized as interacting tissues, not only mechanically, but also in reason of their biochemical cross-talking. In fact, skeletal muscle is the effector of exercise, and movement of sarcomeres and sarcomere-associated structures results in the generation of biochemical signals directed to the myofibers as well as distant tissues [38].

At the same time skeletal muscle is secondarily regulated by biochemical signals released by other tissues (bone, adipose tissue) in response to exercise [39].

In this perspective, the “bone-muscle” unit would be the site of privileged exchanges in which the two tissues communicate via paracrine and endocrine signals to modulate the local microenvironment and distant tissues. The regulation of bone metabolism by muscle occurs through the secretion of specific factors, called myokines [40].

Myostatin is a member of the tumour growth factor-β (TGF-β) family and acts as a negative regulator of muscle mass. Increased levels of myostatin correlate with a state of muscle disuse and sarcopenia. In a similar theme, myostatin negatively impacts bone remodelling, inducing a catabolic, resorptive state, by increasing osteoclastogenesis, and limiting bone formation, via osteoblasts inhibition [41••].

Irisin is one of the newly discovered hormones, resulting from the proteolytic cleavage of fibronectin type III domain 5 (FNDC5), which is secreted mainly by muscular tissue.

Exercise promotes irisin expression and induces anti-inflammatory effects, via downregulation of the Toll-like receptor 4 (TLR4)/myeloid differentiation primary response protein 88 (MyD88) downstream pathway and decrease of NF-kB phosphorylation, leading to a reduced secretion of pro-inflammatory cytokines [42].

As to bone metabolism, irisin promotes osteoblast differentiation through the Wnt-β-catenin pathway and, on the other hand, inhibits osteoclast differentiation and proliferation by suppressing the Receptor Activator of Nuclear Factor κ B ligand (RANKL)/NFATc1 pathway [43].

Reinforcing the strong correlation between myokines and inflammatory response, several ILs are secreted by skeletal muscle, with a wide range of systemic effects.

One of the most significant, interleukin (IL)-6, mostly synthesized by the liver with pro-inflammatory action, could also be released by muscles in response to exercise, inducing anti-inflammatory effects and increasing glucose uptake and insuline-sensitivity [39]. Despite these beneficial effects, the impact of IL-6 on bone is less positive, resulting in the release of RANK by osteoblasts, osteocytes and leukocytes and increases expression of its ligand (i.e. RANK-L) by osteoclasts, finally leading to a net resorptive effect [43].

IL-7 and IL-15 are also strongly related to inflammatory responses and have been shown to be expressed in muscle. IL-7 is a mediator of the acquired immune system, whilst IL-15 is a potent proliferator of innate immune cells. Both demonstrated a strong action on bone resorption, increasing osteoclastogenesis, largely via RANK-L stimulation [23].

Besides myokines, a small number of factors secreted by bone have recently been identified as having effects systemically on a range of tissues, including muscle.

Osteocalcin is a hormone secreted mainly by osteoblasts. Multiple clinical studies have shown that osteocalcin levels increase after exercise, and this has been associated with various metabolic implications with the overall effect of elevated insulin secretion and sensitivity [39]. Indeed, at muscle level, higher osteocalcin concentrations may contribute to an insulin-dependent increase in post-contraction glucose uptake and, from a more functional perspective, to augmented muscle hypertrophy and strength. Counterwise low levels of osteocalcin (e.g. related to inactivity) lead to muscle loss and weakness [42].

Sclerostin, mainly secreted by mature osteocytes, acts as a suppressor of bone formation via the

canonical Wnt/β-catenin pathway. Osteocytes are mechanosensitive cells that coordinate the adaptive response of bone to mechanical stimuli. Hence, sclerostin secretion increases in response to bedrest or unloading, whilst its levels reduce with muscle or bone loading [42].

In conclusion, acute exercise induces the release of pro-anabolic molecules by skeletal muscle and bone, providing effects on both tissues (i.e. determining bone turnover and muscular hypertrophy).

Chronic degenerative conditions such as muscle disuse, atrophy, sarcopenia and ageing are instead associated with the expression of a second set of mediators, which in turn enhance osteoclastogenesis and bone resorption.

### Correlation Between Lean Body Mass and Bone Fragility Induced by HDTs in Cancer Patients

Clinical data supporting the influence of decreased muscle mass on HDT-induced bone fragility mainly derive from studies enrolling subjects with prostate cancer under ADT.

BLADE study was designed to assess changes in body composition (i.e. FBM and LBM by DXA scan) and bone metabolism (i.e. fragments of collagen and BMD) amongst patients affected by non-metastatic prostate cancer receiving an LHRH-antagonist (degarelix) during a period of 12 months [36•].

Twenty-nine patients were enrolled and submitted to DXA scan evaluation and blood test before (baseline) and after (12 months) degarelix administration. At final analysis LBM failed to correlate with markers of bone turnover, both at baseline and after treatment. Changes in bone turnover markers were not influenced by baseline LBM either. Appendicular Lean Mass Index (ALMI), representing the sum of lean tissue in the arms and legs, is considered a more reliable parameter of skeletal muscle mass with respect to LBM, being less influenced by skin, internal organs, tendons, and other non-fatty components [44]. In fact, in further analyses an inverse relationship between ALMI and serum levels of C-terminal telopeptide of type I collagen (CTX, a marker of bone resorption), but not alkaline phosphatase (ALP, a marker of osteoblast activity), was noted at both baseline and after 12 months of degarelix therapy. More importantly, a significant inverse correlation between changes in ALMI and CTX and a direct relationship between changes of ALMI and ALP before and after degarelix were observed. These results support the existence of a functional and biological relationship between muscle and bone tissues and suggest that decrease in lean mass during ADT may influence bone remodelling, leading to bone quality deterioration. As to bone quantity, amongst patients enrolled in BLADE study, ALMI also directly correlated with BMD at total hip but not at lumbar spine, underlying the importance of regional interactions between bone and muscle.

Regional changes in body composition were explored in a further analysis of the BLADE study: LBM was shown to consistently decrease at upper limbs (−4.5%) whilst it did not differ at lower limbs after 12 months of degarelix administration [45•]. This heterogeneity can be explained in light of the different activity of regional muscular masses in elderly subjects (i.e. lower limbs are usually more solicited). Noteworthy, the previously mentioned correlations between LBM and bone turnover biomarkers were preserved even considering regional changes in muscular mass.

Taken together, these data are consistent with published report describing skeletal muscle mass as a causative factor for 15 to 20% of BMD variability at femoral neck in elderly men [46]. The influential role of LBM on bone fragility in this group of patients prompted the design of prospective controlled studies, aiming to evaluate whether supervised physical exercise could be a promising strategy to counter or mitigate changes in body composition and bone metabolism during ADT. The results of these studies, summarized in a recently published meta-analysis, showed that exercise is effective in reducing FBM and increasing LBM; however, no significant effect on BMD was observed [47]. These data highlight the need to find the optimal type of exercise with respect to bone health improvement. Also, BMD may not be a suitable end point for assessing the effectiveness of physical activity since it was shown to be an insensitive parameter as well as poorly associated with HDT-induced bone fragility. Based on the results obtained from BLADE study, bone turnover markers (i.e. CTX) could be more suitable end points.

There are few published data regarding the relationship between LBM and bone health in post-menopausal women undergoing AIs. A South African cross-sectional study found that lean mass index (LMI), and not fat mass, was independently associated with baseline osteoporosis [48]. This study, however, did not provide data on the prospective effect of LMI on AI-induced osteoporosis. In another large retrospective cross-sectional study LBM alone failed to demonstrate any association with VFs prevalence in either the AI-naive or AI-treated subgroups [18•]. With respect to the effect of physical exercise on bone health, four prospective studies involving breast cancer patients were conducted. In two studies only a minority of patients were submitted to AI treatment, providing limited information about the quest of this review paper. In another study physical exercise was associated with an increase in LBM and a decrease in FBM; BMD was maintained amongst exercisers whilst bone loss was observed amongst usual care participants [49]. In the other two randomized studies physical exercise ameliorated body composition parameters; however, BMD was not significantly affected [50,51]. Finally, in a single-arm cross-sectional study, physical activity was inversely correlated with changes in bone turnover biomarkers, indirectly supporting the interaction between muscle mass and bone metabolism [52]. Taken together, the above discussed clinical data may confirm the existence of an interplay between muscular and bone tissues in patients submitted to HDTs, with the highest evidence in favour of reduced bone turnover markers in the context of an increased appendicular muscle mass (i.e. ALMI). Whether physical exercise is protective or not on bone fracture risk is less clearly demonstrated.

Further prospective data correlating the incidence of clinical and morphometric fractures with specific training programmes are needed.

## Interactions Between Adipose and Muscle Tissues in Favouring Bone Fragility: the Osteosarcopenic Obesity

As above described, adipose and muscular tissues are both involved in the regulation of bone turnover, with different mechanisms. Moreover, skeletal muscle is also the final target of many metabolic alterations caused by adipocyte hyperplasia and hypertrophy. One of the most extensively described pathways of fat-muscle cross-talk is represented by the induction of an inflammatory microenvironment through local recruitment of immune cells (such as M1-type macrophages, mast cells, Th1 and other cytokine-secreting cells) and contextual production of pro-inflammatory adipokines, such as leptin and several interleukins, by activated adipocytes [53,54,55].

In addition, adipose tissue is characterized by excessive production and disturbed capacity to store lipids, which accumulate ectopically inside skeletal muscles [56].

Intramuscular lipids and their derivates induce mitochondrial dysfunction characterized by impaired β-oxidation capacity and increased ROS formation, providing lipotoxic environment and insulin resistance. In response to oxidative stress, muscles produce pro-inflammatory myokines contributing to chronic low-grade systemic inflammation [53].

Obesity induced inflammation is also associated with age-related muscle wasting and sarcopenia, skeletal muscle disuse and catabolism: for instance, chronically elevated IL-6 activates the JAK/STAT3 pathway leading to skeletal muscle atrophy [57]. In fact, in older adults, increased plasma levels of interleukins and low-grade inflammation are associated with loss in muscle strength.

Overall obesity sets a vicious cycle in which disrupted skeletal muscle is at the same time target and driver of self-maintained chronic inflammation, in a condition called sarcopenic obesity [58].

Both the above-mentioned molecular underpinnings and clinical observations contribute to define a complex anatomic/functional unit which integrates adipose tissue, muscles and bone.

**Fig. 3:**
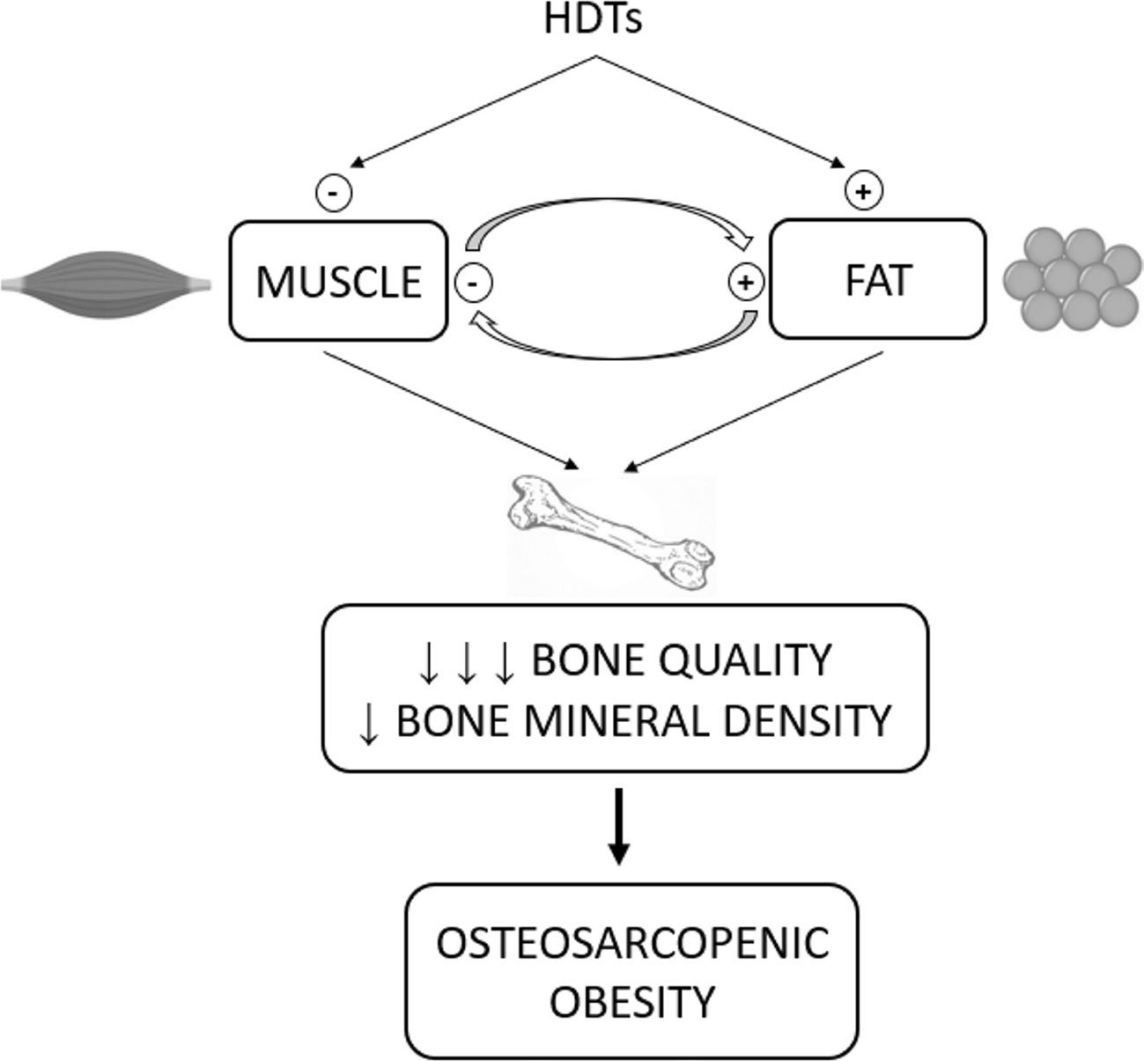
The vicious circle between muscle and fat, enhanced by hormonal deprivation therapies, results in bone quality and bone mineral density alterations, leading to the so-called osteosarcopenic obesity. HDTs hormonal deprivation therapies

Whilst in healthy individuals the interplay amongst the single components is part of physiological ageing, considering cancer patients treated with HDTs the resulting effect is a similar, but more pronounced and accelerated process, leading to sarcopenia, osteoporosis and increased fat mass (the so called osteosarcopenic obesity) [59] (Fig. 3).

Clinical experiences investigating osteosarcopenic obesity in cancer patients are scarce. A cross-sectional clinical study enrolling post-menopausal breast cancer patients who were either AI-naïve or AI-treated sought to evaluate the role of LBM and the interaction between LBM and FBM in predicting the occurrence of VFs [18•]. No interaction was seen between VFs incidence, LBM and AI treatment, whilst a significant interaction was found when considering both LBM and FBM alongside AI therapy. In detail, HDT is associated with the highest rate of new VFs in women presenting with LBM below and FBM above or equal to median values calculated in the same sample.

This evidence suggests that the net effect of HDTs on bone health could be exerted by a shared mechanism in which both adipose and muscular tissues take part.

## Discussion

Clinical management of subjects at higher risk for fracture is well established and described in international guidelines [9,37]. However, data supporting risk assessment and the adoption of preventive strategies mainly come from studies on post-menopausal women, and the extrapolation to cancer patients receiving HDTs could hide some pitfalls. In fact, endocrine derangements induced by cancer treatments are much deeper and more rapid than those observed in menopausal setting, involving tissues other than bone, in a complex interplay which finally leads to extensive body composition alterations.

This review paper focused on the preclinical rationale and initial clinical data supporting a significant role for fat and lean (muscular) body mass variations in determining bone fragility during HDTs in patients with breast and prostate cancer.

As to fat mass, adipose tissue can interfere with bone metabolism through endocrine and paracrine signals, as well as by means of self-maintained chronic inflammation, leading to both bone resorption and impaired bone quality. Counterwise, muscular mass has a trophic role on bone tissue, so that profound alterations in bone quantity and quality are often seen in sarcopenic subjects.

Whilst in non-treated men and women physiologic oestrogen levels do mitigate body composition variations and their relative consequence on bone health, cancer patients under HDTs undergo an extreme condition of hormonal annihilation leading to increased adiposity, loss of muscular mass and enhanced bone fragility.

Though a mechanistic basis supporting this phenomenon in a condition of hormonal deprivation is well established, clinical data on patients under HDTs are currently limited.

Higher fat mass is associated with an excess in morphometric fractures in women [19] and men [13•] under HDTs but failed to correlate with changes in bone turnover biomarkers [36•]. Turning to muscular mass, an inverse correlation was observed between ALMI and bone turnover biomarkers, whilst no evidence of any alteration in BMD or fracture risk in response to LBM variations could be described [36•].

Finally, a causative role of higher fat mass and lower lean mass (i.e. sarcopenic obesity) in the deterioration of bone health during HDTs can be supported. However, these data are limited by the absence of a global analysis focused on the simultaneous effect of fat and lean mass variations, as well as a strong common clinical endpoint (e.g. incidence of new morphometric fractures).

Along this line, convincing clinical evidence in favour of an adipose-muscular interplay on bone fragility is coming from a single-centre, cross-sectional study and suggests that in a hormone-deprived context the higher incidence of VFs would be observed in subjects with higher fat and lower lean mass [18•].

The described increase in bone fragility linked to body composition alterations may be explained through bone quality rather than bone quantity impairment, as suggested by the observed marginal role of BMD in predicting fracture risk for patients undergoing HDTs [7••].

If this interaction is confirmed, it would be reasonable to add sarcopenic obesity to other known risk factors for bone fragility, possibly with the greatest impact for cancer patients under HDTs, leading to a new clinical condition called osteo-sarcopenic obesity.

Furthermore, besides bone fragility, sarcopenic obesity is associated with the risk of cardiovascular events in the general population, being possibly implicated in HDTs induced cardiotoxicity as well [60]. This assumption support sarcopenic obesity as a common target for the prevention of both HDTs associated skeletal and cardiovascular complications.

An important issue in these patients is how to identify and follow-up fat and lean mass alterations, in order to prompt adequate therapeutic approaches, such as the use of bone resorptive agents and lifestyle modifications. Since BMI, though extensively applied, does not take into account the individual proportion of LBM and FBM [61], other more precise and immediate parameters are warranted. DXA scan is a simple diagnostic tool that can provide accurate information on either body composition or bone fragility. Parameters that can be evaluated with DXA go far beyond BMD and include total and district distribution of LBM and FBM, assessment of visceral adipose tissue (VAT) and ALMI as a measure of upper and lower limb muscle mass, TBS and other measures of bone strength and bone geometry [62].

Therapeutic approaches to sarcopenic obesity could counteract bone fragility and cardiovascular complications in cancer patients receiving HDTs, representing a relevant matter of research.

To now, physical activity is considered the most effective intervention, given its many biological effects, such as the reduction of oxidative stress, the increase in number and size of muscle fibres, the promotion of insulin sensitivity and the downregulation of systemic inflammatory biomarkers [63]. Available data suggest that aerobic training improves cardiorespiratory fitness, mostly counteracting obesity, whilst resistance training is associated with increase or maintenance of muscle strength and flexibility [64] as well as improvement in BMD [65]. Heterogeneity in body composition changes, described in prostate cancer patients during ADT, provides a rationale for tailored supervised physical activity based on individual variations of FBM and LBM by DXA scan monitoring [45•].

## Conclusion

HDTs induce profound physical and metabolic derangements, involving adipose and muscular tissues: accumulating evidence support these alterations to be regarded as the most prominent mechanism of toxicity, leading to bone fragility as well as cardiovascular disease.

Targeting osteosarcopenic obesity for the prevention and treatment of HDTs-related metabolic and skeletal complications is promising and worth further research.
